# Supplementary material for: Diagnostic accuracy of point-of-care ultrasound for pulmonary tuberculosis: A systematic review
Source: PLoS One. 2021 May 7;16(5):e0251236. doi: 10.1371/journal.pone.0251236 (PMC8104425; doi:10.1371/journal.pone.0251236)
Supplement: S1 Appendix — (PDF) [file pone.0251236.s001.pdf]

S1 Appendix: Search Strategies

Limits: Jan 1, 2010-
English or French

Platform	Database(s)	Database coverage dates	# Results	Search Date
OvidSP	Ovid MEDLINE ALL(R)	1946 -	1209	2020/06/01
OvidSP	EMBASE	1996 -	709	2020/06/01
Web of Science	SCI-EXPANDED, ESCI	1900 -	665	2020/06/01
Cochrane Library	CENTRAL (Trials)	Inception -	232	2021/02/10
Scopus		Inception-	3133	2020/06/01
	**TOTAL NUMBER OF RECORDS**		5879	

Number of records after duplicates removed: 3919 records

MEDLINE (Ovid), June 1, 2020
1209 records
Database: Ovid MEDLINE(R) ALL <1946 to May 29, 2020>
Search Strategy:
----
1 exp tuberculosis/ or mycobacterium tuberculosis/ (211746)
2 tuberculo*.cl,mp,so. or (tb or ptb).ti,kf. (264635)
3 1 or 2 (265038)
4 exp ultrasonography/ (434551)
5 (echogra* or echoscop* or echosound* or ultraso* or sonogra* or sonolog* or POCUS).cl,mp,so. (513962)
6 4 or 5 (646410)
7 diagnosis/ or predictive value of tests/ or exp "sensitivity and specificity"/ or exp ultrasonography/di, dg (597440)
8 (diagnos* or detect* or gold standard or index test* or inter method* or intermethod* or inter rater or inter reader or point of care or reference standard* or reproducib* or screen* or triag* or sensitiv* or positive* or negative* or specific* or accura* or roc or "area under the curve").cl,mp,so. or (assess* or compar* or evaluat* or feasib* or finding* or utility or valid*).cl,ti,so. (12730590)
9 7 or 8 (12755581)
10 3 and 6 and 9 (2748)
11 ((tuberculo* or tb or ptb or infectious disease* or communicable disease*) and (echogra* or echoscop* or echoso* or ultraso* or sonogra* or sonolog* or POCUS)).ti,kf. (544)
12 10 or 11 (2860)
13 limit 12 to yr="2010 -Current" (1302)
14 limit 13 to (english or french) (1209)

****

Embase (Ovid), June 1, 2020

709 records
Database: Embase <1996 to 2020 Week 22>
Search Strategy:
----
1 exp tuberculosis/ or *mycobacterium tuberculosis/ or tuberculo*.ti,ab,kw. or (tb or ptb).ti,kw. (188046)
2 exp *echography/ or (echogra* or echoscop* or echosound* or ultraso* or sonogra* or sonolog* or POCUS).ti,kw. (292289)
3 diagnosis/ or diagnostic accuracy/ or diagnostic test accuracy study/ or point of care system/ or predictive value/ or reliability/ (1138949)
4 (diagnos* or detect* or gold standard or index test* or inter method* or intermethod* or inter rater or inter reader or point of care or reference standard* or reproducib* or screen* or triag* or sensitiv* or positive* or negative* or specific* or accura* or roc or "area under the curve").ti,ab,kw. or (assess* or compar* or evaluat* or feasib* or finding* or utility or valid*).ti,kw. (11191098)
5 3 or 4 (11292169)
6 1 and 2 and 5 (1354)
7 ((tuberculo* or tb or ptb or infectious disease* or communicable disease*) and (echogra* or echoscop* or echosound* or ultraso* or sonogra* or sonolog* or POCUS)).ti,kw. (627)
8 6 or 7 (1447)
9 limit 8 to yr="2010 -Current" (1059)
10 limit 9 to (english or french) (994)
11 10 not (conference abstract or conference paper).pt. (709)

****

Science Citation Index – Expanded (SCI-EXPANDED), Emerging Sources Citation Index (ESCI) (Web of Science), June 1, 2020
665 records
(
(TS=(tuberculo*) OR TI=(tb OR ptb) OR AK=(tb OR ptb) OR KP=(tb OR ptb))
AND
(
TI=(echogra* OR echoscop* OR echosound* OR ultraso* OR sonogra* OR sonolog* OR POCUS) OR AK=(echogra* OR echoscop* OR echosound* OR ultraso* OR sonogra* OR sonolog* OR POCUS) OR KP=(echogra* OR echoscop* OR echosound* OR ultraso* OR sonogra* OR sonolog* OR POCUS)
)
AND
(
TS=(diagnos* OR detect* OR "gold standard" OR "index test*" OR "inter method*" OR intermethod* OR "inter rater" OR "inter reader" OR "point of care" OR "reference standard*" OR reproducib* OR screen* OR triag* OR sensitiv* OR positive* OR negative* OR specific* OR accura* OR roc OR "area under the curve") OR TI=(assess* OR compar* OR evaluat* OR feasib* OR finding* OR utility OR valid*) OR AK=(assess* OR compar* OR evaluat* OR feasib* OR finding* OR utility OR valid*) OR KP=(assess* OR compar* OR evaluat* OR feasib* OR finding* OR utility OR valid*)
)
)
OR
(
TI=((tuberculo* OR tb OR ptb OR "infectious disease*" OR "communicable disease*") AND (echogra* OR echoscop* OR echoso* OR ultraso* OR sonogra* OR sonolog* OR POCUS)) OR AK= ((tuberculo* OR tb OR ptb OR "infectious disease*" OR "communicable disease*") AND (echogra* OR echoscop*

OR echoso* OR ultraso* OR sonogra* OR sonolog* OR POCUS)) OR KP= ((tuberculo* OR tb OR ptb OR "infectious disease*" OR "communicable disease*") AND (echogra* OR echoscop* OR echoso* OR ultraso* OR sonogra* OR sonolog* OR POCUS))

)
Limits:
2010-
English OR French
SCI-EXPANDED, ESCI
****

Scopus, June 1, 2020
3133 records

(
(
(

TITLE-ABS-KEY(tuberculo*) OR TITLE(tb or ptb) OR KEY(tb or ptb)

)

AND

(

TITLE(echogra* or echoscop* or echosound* or ultraso* or sonogra* or sonolog* or POCUS) OR KEY(echogra* or echoscop* or echosound* or ultraso* or sonogra* or sonolog* or POCUS)

)

AND

(
TITLE-ABS-KEY(diagnos* or detect* or "gold standard" or "index test*" or "inter method*" or intermethod* or "inter rater" or "inter reader" or "point of care" or "reference standard*" or reproducib* or screen* or triag* or sensitiv* or positive* or negative* or specific* or accura* or roc or "area under the curve") or TITLE(assess* or compar* or evaluat* or feasib* or finding* or utility or valid*) OR KEY(assess* or compar* or evaluat* or feasib* or finding* or utility or valid*)
)
)
OR

(
TITLE((tuberculo* or tb or ptb or "infectious disease*" or "communicable disease*") and (echogra* or echoscop* or echoso* or ultraso* or sonogra* or sonolog* or POCUS)) OR KEY ((tuberculo* or tb or ptb or "infectious disease*" or "communicable disease*") and (echogra* or echoscop* or echoso* or ultraso* or sonogra* or sonolog* or POCUS))
)
)
AND PUBYEAR AFT 2009 AND LANGUAGE(English or French)

****

Cochrane Central Register of Controlled Trials (Cochrane Library), June 1, 2020
232 records
Search Name:
Date Run: 10/02/2021 16:38:52
Comment:

ID	Search	Hits
#1	(tuberculo* OR tb OR ptb):ti,ab,kw OR (infectious next disease* OR communicable next disease*):ti,kw	
#2	(echogra* OR echoscop* OR echosound* OR ultraso* OR sonogra* OR sonolog* OR POCUS):ti,ab,kw	
#3	#1 AND #2 with Publication Year from 2010 to 2020, in Trials	232
