# Supplementary material for: Diagnostic accuracy of point-of-care ultrasound for pulmonary tuberculosis: A systematic review
Source: PLoS One. 2021 May 7;16(5):e0251236. doi: 10.1371/journal.pone.0251236 (PMC8104425; doi:10.1371/journal.pone.0251236)
Supplement: S2 Appendix — (PDF) [file pone.0251236.s002.pdf]

S2 Appendix: Data Extraction Form

Data extractor					
First author					
Corresponding author					
Corresponding author email					
Title of paper					
Language of paper					
Publication date					
**1. STUDY DETAILS**	**Highlight answers with a colourful background**				
a. Type of study	Quantitative observational study				
	Mixed methods study with a quantitative component				
	Intervention study				
	Unclear/not reported				
b. Study data collection	Prospective				
	Retrospective				
	Unclear/not reported				
c. Participant selection	Convenience				
	Consecutive				
	Random				
	Unclear/not reported				
	Other (specify in box to the right)				
d. Study country					
e. Study region					
f. Country income status	Low				
	Middle				
	High				
**2. SETTING AND TEST DETAILS**					
a. Healthcare setting	Primary care				
	Regional hospital				
	Tertiary hospital				
	Other (specify in box to the right)				
b. Type of ultrasound used	Coventional				
	POCUS				
	Unclear/not reported				
c. Type of tuberculosis	PTB				
	Miliary TB				
	EPTB				
	Unclear/not reported				
	Other (specify in box to the right)				
d. Purpose of test	Screening				
	Diagnosis				
	Unclear/not reported				
e. Type of healthcare provider	Physician				
	Nurse				
	Radiographer/technologist				
	Other (specify in box to the right)				
	Unclear/not reported				
**3. PATIENT DEMOGRAPHICS**	**Questions in bold must be answered**	Overall	Confirmed TB	Unconfirmed TB	Unlikely/non-TB
	**Data available for category? (yes/no)**				
	Number of included patients				
	Percent female				
	Median age in years (IQR)				
	Mean age in years (SD)				
	Age range				
	**Children younger than 15 included? (yes/no/unclear)**				
	Percent children younger than 15				
	Median child age in months (IQR)				
	Median child age in years (IQR)				
	Mean child age in months (SD)				
	Mean child age in years (SD)				
	**HIV patients included? (yes/no/unclear)**				
	If yes, what percentage?				
	**Past history of TB? (yes/no/unclear)**				
	If yes, what percentage?				
	**Patients on TB treatment included? (yes/no/unclear)**				
	If yes, what percentage?				

4. SPECIMEN TYPES FOR REFERENCE TEST

If no detailed breakdown, indicate which specimen types were used

	Number of specimens	Percentage
Sputum		
Brochoalveolar lavage		
Endotracheal aspirate		
Other (specify below)		

5. INDEX TEST

a. Were the ultrasound results interpreted without knowledge of the results of the reference standard?

Yes
No
Unclear/not reported

6. REFERENCE STANDARD

i. Reference standard for adults

a. Reference standard used

Solid culture	LJ
	7H10
	7H11
	Other (specify right)
Liquid culture	MGIT
	Bactec 460
	Other (specify right)
Molecular test	Xpert
	Line probe assay (specify right)
	TB-LAMP (specify right
	Other (specify right)
Imaging standard	CXR
	CT
	MRI
	Other (specify right)
Other standard	(Specify right)

b. Did all patients receive a reference standard?

Yes
No
Unclear/not reported
If no, why not? (specify right)

c. Did all patients receive the same reference standard?

Yes
No
Unclear/not reported
If no, why not? (specify right)

d. Were the reference standard results interpreted without knowledge of the results of the ultrasound?

Yes
No
Unclear/not reported

e. What was the time period between the ultrasound scan and the performance of the reference standard test?

Specify right
Unclear/not reported

ii. Reference standard for children

a. Reference standard used	Solid culture (specify right)
	Liquid culture (specify right)
	Molecular test (specify right)
	Imaging reference standard (specify right)
	Response to treatment reference standard
	i. Specify treatment
	ii. Specify expected response
	iii. Specify time period over which response was monitored
	Composite reference standard (specify right)
	Clinical reference standard (specify right)
	Other reference standard (specify right)

b. Did all patients receive a reference standard?

Yes
No
Unclear/not reported
If no, why not? (specify right)

c. Did all patients receive the same reference standard?

Yes
No
Unclear/not reported
If no, why not? (specify right)

d. Were the reference standard results interpreted without knowledge of the results of the ultrasound?
Yes
No
Unclear/not reported

e. What was the time period between the ultrasound scan and the performance of the reference standard test?
Specify right
Unclear/not reported

7. ANALYSIS
a. Were all patients included in the analysis?
Yes
No
Unclear/not reported

8. RESULTS
A. Adults
i. Higher-quality reference standard (liquid or solid culture or molecular test)
a. True positives, false positives, true negatives, false negatives:
Not reported

			Reference standard result		
	Ultrasound finding (e.g. consolidation)	**Ultrasound result**	Positive	Negative	**Total**
1		Positive			
		Negative			
		**Total**			
2		Positive			
		Negative			
		**Total**			
3		Positive			
		Negative			
		**Total**			
4		Positive			
		Negative			
		**Total**			
5		Positive			
		Negative			
		**Total**			
6		Positive			
		Negative			
		**Total**			
7		Positive			
		Negative			
		**Total**			
8		Positive			
		Negative			
		**Total**			

(add more rows if required)

Specify reference standard
Liquid culture
Solid culture
Molecular test

b. Area under the curve
Specify right
Not reported

ii. Lower-quality reference standard (correlation with another imaging modality)
a. True positives, false positives, true negatives, false negatives:
Not reported

			Reference standard result		
	Ultrasound finding (e.g. consolidation)	**Ultrasound result**	Positive	Negative	**Total**
1		Positive			
		Negative			
		**Total**			
2		Positive			
		Negative			
		**Total**			
3		Positive			
		Negative			
		**Total**			
4		Positive			
		Negative			
		**Total**			
5		Positive			
		Negative			
		**Total**			
6		Positive			
		Negative			

	**Total**			
7	Positive			
	Negative			
	**Total**			
8	Positive			
	Negative			
	**Total**			

(add more rows if required)

Specify reference standard — CXR
CT
MRI
Other (specify right)

b. Area under the curve — Specify right
Not reported

iii. Kappa score for reliability
a. Kappa score for **inter**-rater reliability
Not reported

Ultrasound finding	Kappa score

(add more rows if required)

b. Kappa score for **intra**-rater reliability
Not reported

Ultrasound finding	Kappa score

(add more rows if required)

B. Children
i. Validity **(if multiple reference standards used, complete multiple tables)**
a. True positives, false positives, true negatives, false negatives:
Not reported

	Ultrasound finding (e.g. consolidation)		**Reference standard result**		
		**Ultrasound result**	Positive	Negative	**Total**
1		Positive			
		Negative			
		**Total**			
2		Positive			
		Negative			
		**Total**			
3		Positive			
		Negative			
		**Total**			
4		Positive			
		Negative			
		**Total**			
5		Positive			
		Negative			
		**Total**			
6		Positive			
		Negative			
		**Total**			
7		Positive			
		Negative			
		**Total**			
8		Positive			
		Negative			
		**Total**			

Specify reference standard — Liquid culture
Solid culture
Molecular test
Imaging
Composite
Clinical
Response to treatment
Other (specify right)

b. Area under the curve — Specify right
Not reported

Data for second reference standard (if required)

a. True positives, false positives, true negatives, false negatives:

Not reported

Ultrasound finding (e.g. consolidation)

		Reference standard result		
	**Ultrasound result**	Positive	Negative	**Total**
1	Positive			
	Negative			
	**Total**			
2	Positive			
	Negative			
	**Total**			
3	Positive			
	Negative			
	**Total**			
4	Positive			
	Negative			
	**Total**			
5	Positive			
	Negative			
	**Total**			
6	Positive			
	Negative			
	**Total**			
7	Positive			
	Negative			
	**Total**			
8	Positive			
	Negative			
	**Total**			

Specify reference standard
- Liquid culture
- Solid culture
- Molecular test
- Imaging
- Composite
- Clinical
- Response to treatment
- Other (specify right)

b. Area under the curve
- Specify right
- Not reported

iii. Kappa score for reliability

a. Kappa score for **inter**-rater reliability

Not reported

Ultrasound finding	Kappa score

(add more rows if required)

b. Kappa score for **intra**-rater reliability

Not reported

Ultrasound finding	Kappa score

(add more rows if required)

9. QUADAS-2

DOMAIN 1: PATIENT SELECTION

A. Risk of Bias

Was a consecutive or random sample of patients enrolled?
- Yes
- No
- Unclear

Was a case-control design avoided?
- Yes
- No
- Unclear

Did the study avoid inappropriate exclusions?
- Yes
- No
- Unclear

Could the selection of patients have introduced bias?
- **LOW RISK**
- **HIGH RISK**
- **UNCLEAR RISK**

B. Concerns regarding applicability

Is there concern that the included patients do not match the review question?	**LOW CONCERN**<br>**HIGH CONCERN**<br>**UNCLEAR CONCERN**

DOMAIN 2: INDEX TEST(S)

If more than one index test was used, please complete for each test.

A. Risk of Bias

Were the index test results interpreted without knowledge of the results of the reference standard?	Yes<br>No<br>Unclear
If a threshold was used, was it pre-specified	Yes<br>No<br>Unclear
**Could the conduct or interpretation of the index test have introduced bias?**	**LOW RISK**<br>**HIGH RISK**<br>**UNCLEAR RISK**

B. Concerns regarding applicability

Is there concern that the index test, its conduct, or interpretation differ from the review question?	**LOW CONCERN**<br>**HIGH CONCERN**<br>**UNCLEAR CONCERN**

DOMAIN 3: REFERENCE STANDARD

A. Risk of Bias

Is the reference standard likely to correctly classify the target condition?	Yes<br>No<br>Unclear
Were the reference standard results interpreted without knowledge of the results of the index test?	Yes<br>No<br>Unclear
**Could the reference standard, its conduct, or its interpretation have introduced bias?**	**LOW RISK**<br>**HIGH RISK**<br>**UNCLEAR RISK**

B. Concerns regarding applicability

Is there concern that the target condition as defined by the reference standard does not match the review question?	**LOW CONCERN**<br>**HIGH CONCERN**<br>**UNCLEAR CONCERN**

DOMAIN 4: FLOW AND TIMING

A. Risk of Bias

Was there an appropriate interval between index test(s) and reference standard?	Yes<br>No<br>Unclear
Did all patients receive a reference standard?	Yes<br>No<br>Unclear
Did patients receive the same reference standard?	Yes<br>No<br>Unclear
Were all patients included in the analysis?	Yes<br>No<br>Unclear
Could the patient flow have introduced bias?	**LOW RISK**<br>**HIGH RISK**<br>**UNCLEAR RISK**

complete only if paper had reproducibility data

Additional: REPRODUCIBILITY

Was the time interval between the repeated tests appropriate?	Yes<br>No<br>Unclear
Were the test conditions similar for the repeated tests (type of administration, environment, instructions)?	Yes<br>No<br>Unclear
Was a Kappa score calculated?	Yes<br>No<br>Unclear

Could the reproducibility data be biased?	**LOW RISK**<br>**HIGH RISK**<br>**UNCLEAR RISK**
