# Supplementary material for: Diagnostic accuracy of point-of-care ultrasound for pulmonary tuberculosis: A systematic review
Source: PLoS One. 2021 May 7;16(5):e0251236. doi: 10.1371/journal.pone.0251236 (PMC8104425; doi:10.1371/journal.pone.0251236)
Supplement: S3 Appendix — (PDF) [file pone.0251236.s003.pdf]

S3 Appendix: Additional reproducibility domain for quality assessment

(1) Was the time interval between the repeated tests appropriate? [Yes/No/Unclear]
(2) Were the test conditions similar for the repeated tests (type of administration, environment, instructions)? [Yes/No/Unclear]
(3) Was a Kappa score calculated? [Yes/No/Unclear].

Like the risk of bias questions in QUADAS-2, the answer to the question “Could the reproducibility data be biased?” [Low risk/High risk/Unclear risk] was based on the answers to the three reproducibility criteria questions.

Based on the work of Mokkink et al. (2018) (28).
