# Supplementary material for: Diagnostic accuracy of point-of-care ultrasound for pulmonary tuberculosis: A systematic review
Source: PLoS One. 2021 May 7;16(5):e0251236. doi: 10.1371/journal.pone.0251236 (PMC8104425; doi:10.1371/journal.pone.0251236)
Supplement: S4 Appendix — (PDF) [file pone.0251236.s004.pdf]

Domain 1: Patient Selection

Risk of Bias: Could the selection of patients have introduced bias?

We will answer 'high risk' if any of the signalling questions have the answer 'no', 'low risk' if all of the signalling questions have the answer 'yes' and 'unclear risk' if one or more of the signalling questions has the answer 'unclear' and the other questions have the answer 'yes'.

Signaling question 1: Was a consecutive or random sample of patients or specimens enrolled?

o We will answer 'yes' if the study enrolled a consecutive or random sample of eligible patients; 'no' if the study selected patients by convenience, and 'unclear' if the study did not report the manner of patient selection or this cannot be discerned.

Signaling question 2: Was a case-control design avoided?

o We will answer 'yes' if a case-control study design is not used or the results of suspected pulmonary TB (PTB) cases were not compared to healthy individuals. We will score 'no' if a case-control study design was used and results of suspected PTB cases were compared to healthy individuals, or if the study only included confirmed cases of PTB. We will score 'unclear' if the study did not report the design or this cannot be discerned.

Signaling question 3: Did the study avoid inappropriate exclusions?

o We will answer 'yes' if inappropriate exclusions were avoided. We will answer 'no' if studies note specific inappropriate exclusions. Inappropriate exclusions include those based on ultrasound scans considered 'difficult to read'; those based on the arbitrary exclusion of patients based on their age; those based on the arbitrary exclusion of patients based on their comorbidities or other infections; and those based on prior knowledge of results of TB testing. We will answer 'unclear' if exclusions were not clearly reported but there is suspicion that inappropriate exclusions occurred.

Applicability: Are there concerns that the included patients and setting do not match the review question?

o We are interested in how the index test (ultrasound) performed in diagnosing or screening PTB. We will judge 'low concern' if the ultrasound was performed on patients with suspected PTB (using any definition of suspected PTB) in any healthcare setting in which patients are typically screened for or diagnosed with PTB (e.g. primary care setting, regional hospital, referral hospital, healthcare centres associated with the National Tuberculosis Programmes), irrespective of the TB burden of the country. We will judge 'high concern' if the enrolled patients do not match the scenario described above, or if only confirmed TB cases were included. We will judge 'unclear concern' if we cannot tell.

Domain 2: Index Test

Risk of Bias: Could the conduct or interpretation of the index test have introduced bias?

We will answer 'high risk' if any of the signalling questions have the answer 'no', 'low risk' if all of the signalling questions have the answer 'yes' and 'unclear risk' if one or more of the signalling questions has the answer 'unclear' and the other questions have the answer 'yes'.

Signaling question 1: Were the index test results interpreted without knowledge of the results of the reference standard?

o We will answer 'yes' only if it was explicitly stated that the ultrasound results were interpreted without knowledge of the results of the reference standard, or if it was clear by the timing of the tests that the reference standard results could not have been known when the index test was interpreted; 'no' if the ultrasound results were interpreted with knowledge of the results of

the reference standard, or if only confirmed TB cases were included; and 'unclear' if the study did not report the blinding of the ultrasound interpreters or this cannot be discerned.

Signaling question 2: If a threshold was used, was it prespecified?

o We will answer 'yes' if the study states the use of a single, pre-specified, cut-off value for each lung ultrasound sign for which it is appropriate (e.g. "subpleural, nodular, hypoechoic region < 1 × 1 cm, with distinct borders and trailing comet-tail artifacts") and the study pre-specifies how each non-numerical sign was defined (e.g. "diffuse, bilateral pattern of multiple B-lines and subpleural sonographic granularity"). We will answer 'no' if multiple cut-off values or sign definitions were evaluated for any one sign and an optimal one was subsequently chosen based on maximising test accuracy. We will judge 'unclear' if any one cut-off value or sign definition was used but not reported or if we cannot tell.

Applicability: Are there concerns that the index test, its conduct, or its interpretation differ from the review question?

o We will judge 'low concern' if study participants were given an ultrasound scan of the lungs, delivered by any kind of healthcare provider, and the ultrasound signs that were looked for (e.g. consolidation) were reported. We will judge 'high concern' if the ultrasound signs looked for were not reported. We will judge 'unclear concern' if we cannot tell.

Domain 3: Reference Standard

Risk of Bias: Could the reference standard, its conduct, or its interpretation have introduced bias?
We will answer 'high risk' if any of the signalling questions have the answer 'no', 'low risk' if all of the signalling questions have the answer 'yes' and 'unclear risk' if one or more of the signalling questions has the answer 'unclear' and the other questions have the answer 'yes'.

Signaling question 1: Is the reference standard likely to correctly classify the target condition?

For adults:

o We will answer 'yes' if all study participants were given a reference standard test of liquid or solid culture or a molecular test. We will answer 'no' if not all study participants were given a reference standard test of liquid or solid culture or a molecular test. We will answer 'unclear' if we cannot tell.

For children:

o We will answer 'yes' if all study participants were given a reference standard test of any type (CT, MRI, CXR, liquid culture, solid culture, molecular test, clinical reference standard, response to treatment reference standard, composite reference standard including any of the above). We will answer 'no' if some study participants were not given a reference standard test. We will answer 'unclear' if we cannot tell.

Signaling question 2: Were the reference standard results interpreted without knowledge of the results of the index test?

o We will answer 'yes' if blinding was explicitly stated, or if it was clear by the timing of the tests that the reference standard results could not have been known when the index test was interpreted. We will answer 'no' if the study stated that the reference standard was interpreted with knowledge of the index test result. We will score 'unclear' if this was not stated or was addressed inadequately.

Applicability: Are there concerns that the target condition as defined by the reference standard does not match the question?

For adults:

o We will answer ‘low concern’ if all study participants were given a reference standard test of liquid or solid culture or a molecular test. We will answer ‘high concern’ if not all study participants were given a reference standard test of liquid or solid culture or a molecular test. We will answer ‘unclear concern’ if we cannot tell.

o Additionally, in cases where the reference standard test was liquid or solid culture alone, we will answer ‘low concern’ if speciation was performed for positive cultures, we will answer ‘high concern’ if no speciation was performed and we will judge ‘unclear concern’ if we cannot tell.

For children:

o We will ‘low concern’ if all study participants were given a reference standard test of any type (CT, MRI, CXR, liquid culture, solid culture, molecular test, clinical reference standard, response to treatment reference standard, composite reference standard including any of the above). We will answer ‘high concern’ if all study participants were not given a reference standard test. We will answer ‘unclear concern’ if we cannot tell.

o Additionally, in cases where the reference standard test was liquid or solid culture alone, we will answer ‘low concern’ if speciation was performed for positive cultures, we will answer ‘high concern’ if no speciation was performed and we will judge ‘unclear concern’ if we cannot tell.

Domain 4: Flow and Timing

Risk of Bias: Could the patient flow have introduced bias?

We will answer ‘high risk’ if any of the signalling questions have the answer ‘no’, ‘low risk’ if all of the signalling questions have the answer ‘yes’ and ‘unclear risk’ if one or more of the signalling questions has the answer ‘unclear’ and the other questions have the answer ‘yes’.

Signaling question 1: Was there an appropriate interval between the index test and reference standard?

o We will answer ‘yes’ if the tests were performed within 14 days of each other and both occurred less than 14 days after treatment initiation. We will answer ‘no’ if the reference test and ultrasound scan occurred more than 14 days apart, or either one occurred more than 14 days after treatment initiation. We will answer ‘unclear’ if this was not stated.

Signaling question 2: Did all patients receive a reference standard?

o We will answer ‘yes’ if all participants in the study or a subset of participants in the study (for whom we will extract data) received a reference standard. We will answer ‘no’ if all participants in the study, or in the subset for whom we will extract data, did not receive a reference standard. We will answer ‘unclear’ if we cannot tell.

Signaling question 3: Did all patients receive the same reference standard?

o We will answer ‘yes’ if all participants in the study or a subset of participants in the study (for whom we will extract data) received the same reference standard or reference standards. We will answer ‘no’ if all participants in the study, or in the subset for whom we will extract data, did not receive the same reference standard or reference standards. We will answer ‘unclear’ if we cannot tell.

Signaling question 4: Were all patients included in the analysis?

o The answer to this question will be determined by comparing the number of patients enrolled with the number of patients included in the two-by-two tables. We will note if authors record the number of indeterminate results. We will answer ‘yes’ if the number of participants enrolled was clearly stated and corresponded to the number presented in the analysis or if exclusions were adequately described. We will answer 'no’ if there were participants missing or

excluded from the analysis and there was no explanation given. We will answer 'unclear ' if not enough information was given to assess whether participants were excluded from the analysis.

Additional Domain: Reproducibility

Risk of Bias: Could the reproducibility data be biased? (Complete only if paper had reproducibility data)

We will answer ‘high risk’ if any of the signalling questions have the answer ‘no’, ‘low risk’ if all of the signalling questions have the answer ‘yes’ and ‘unclear risk’ if one or more of the signalling questions has the answer ‘unclear’ and the other questions have the answer ‘yes’.

Signaling question 1: Was the time interval between the repeated tests appropriate?

o We will answer ‘yes’ if the ultrasound scan was performed once, less than 14 days after treatment initiation, by a single operator who saved the images and the images were interpreted separately by at least one other healthcare provider; we will answer ‘yes’ if ultrasound scans were performed more than once by the same operator, so long as the scans occurred within 14 days of each other and both occurred less than 14 days after treatment initiation; we will also answer ‘yes’ if multiple ultrasound scans were performed at separate times by at least two healthcare providers, so long as the scans occurred within 14 days of each other and both occurred less than 14 days after treatment initiation. We will answer ‘no’ if any of the ultrasound scans occurred more than 14 days after treatment initiation or scans occurred more than 14 days apart. We will answer ‘unclear’ if this was not stated.

Signaling question 2: Were the test conditions similar for the repeated tests (type of administration, environment, instructions)?

o We will answer ‘yes’ if the ultrasound scan was performed once by a single operator who saved the images, or if ultrasound scans were performed by different operators at different times, so long as they used the same methodology for the scans (including length of scan and positioning of patient), looked for the same ultrasound signs and reported the results in the same way. We will answer ‘no’ if scans performed by different operators did not use the same methodology, look for the same ultrasound signs or report the results in the same way. We will answer ‘unclear’ if this was not stated.

Signaling question 3: Was a Kappa score calculated?

o We will answer ‘yes’ if a Kappa score was calculated, ‘no’ if no Kappa score was calculated and ‘unclear’ if this was not stated.
