# Supplementary material for: Diagnostic accuracy of point-of-care ultrasound for pulmonary tuberculosis: A systematic review
Source: PLoS One. 2021 May 7;16(5):e0251236. doi: 10.1371/journal.pone.0251236 (PMC8104425; doi:10.1371/journal.pone.0251236)
Supplement: S5 Appendix — (PDF) [file pone.0251236.s005.pdf]

S6 Appendix: Reasons for exclusion at full-text screening

Study	Reason for exclusion
Alici 2015 [1]	Not PTB
Amin 2016 [2]	Not PTB
Andronikou 2017 [3]	Editorial
ASER abstracts 2014 [4]	Not a diagnostic accuracy study
Balaji 2015 [5]	Not PTB
Belard 2014a [6]	Editorial
Belard 2014b [7]	Not a diagnostic accuracy study
Belard 2016 [8]	Review
Belard 2017 [9]	Not PTB
Belard 2019 [10]	Not a diagnostic accuracy study
BenSaad-Baouab 2019 [11]	Not a diagnostic accuracy study
Bhalla 2015 [12]	Review
Brindle 2013 [13]	Not PTB
Brunetti 2016 [14]	Review
Buonsenso 2018 [15]	Editorial
Buonsenso 2019 [16]	Editorial
Burleson 2020 [17]	Not a diagnostic accuracy study
Canan 2018 [18]	Not PTB
Chesov 2018 [19]	Review
DiGennaro 2018 [20]	Review
dos Reis 2018 [21]	Endobronchial ultrasound
Elmahalawy 2017 [22]	Not PTB
Elnaem 2017 [23]	Not PTB
Fentress 2018 [24]	Review
Gati 2018 [25]	Not PTB
Gavrila 2020 [26]	Review
Giordani 2019 [27]	Editorial
Heller 2010a [28]	Not a diagnostic accuracy study
Heller 2010b [28]	Not a diagnostic accuracy study
Heller 2014 [29]	Not PTB
Heller 2017 [30]	Not a diagnostic accuracy study
Henriquez-Camacho 2015 [31]	Review
Heuvelings 2016 [32]	Not a diagnostic accuracy study
Heuvelings 2017a [33]	Duplicate data
Heuvelings 2017b [34]	Duplicate data
Heuvelings 2017c [35]	Duplicate data
Heuvelings 2019 [36]	Duplicate data
Hunter 2016 [37]	Not PTB
Kahn 2020 [38]	Not PTB
Kaminstein 2019 [39]	Review
Mirijello 2019 [40]	Editorial
Montuori 2019 [41]	Editorial
Moseme 2014 [42]	Not a diagnostic accuracy study
Ndege 2019 [43]	Not PTB
Ndege 2020 [44]	Not PTB
Pomykala 2019 [45]	Review
Pool 2017 [46]	Not a diagnostic accuracy study

Van Hoving 2013 [47]	Not a diagnostic accuracy study
Van Hoving 2020 [48]	Not PTB

Note: Heller 2010a and Heller 2010b are the same paper indexed differently so only 48 unique references appear in this table.

1. Alici IO, Demirci NN, Yilmaz A, Karakaya J, Erdogan Y. Starry sky sign: A prevalent sonographic finding in mediastinal tuberculous lymph nodes. Endosc Ultrasound. 2015;4(3):225-8. Epub 2015/09/17. doi: 10.4103/2303-9027.163004. PubMed PMID: 26374581; PubMed Central PMCID: PMCPMC4568635.
2. Amin H. Role of transthoracic ultrasound in the diagnosis of some chest diseases. Egyptian Journal of Chest Diseases and Tuberculosis. 2016;65(4):851-8. doi: https://doi.org/10.1016/j.ejcdt.2016.03.007.
3. Andronikou S, Sergot L. 'Point-of-care ultrasound' - legitimate terminology. Pediatr Radiol. 2017;47(13):1849-50. Epub 2017/11/18. doi: 10.1007/s00247-017-3978-7. PubMed PMID: 29149374.
4. ASER abstracts. Scientific, Educational Abstracts, and Case-of-the-Day Presented at the ASER 2014 Annual Scientific Meeting and Postgraduate Course September 10–13, Portland, Oregon. Emergency Radiology. 2014;21(5):431-71. doi: 10.1007/s10140-014-1261-x.
5. Balaji BS, Kalpana S, Adhisivam B, Venkatesh C, Kapoor A, Sibal A, et al. Correspondence. Indian Pediatrics. 2015;52(8):715-9. doi: 10.1007/s13312-015-0705-z.
6. Bélard S, Andronikou S, Pillay T, Grobusch MP, Zar HJ. New imaging approaches for improving diagnosis of childhood tuberculosis. S Afr Med J. 2014;104(3):181-2. Epub 2014/06/06. doi: 10.7196/samj.7984. PubMed PMID: 24897819.
7. Bélard S, Heller T, Grobusch MP, Zar HJ. Point-of-care ultrasound: a simple protocol to improve diagnosis of childhood tuberculosis. Pediatr Radiol. 2014;44(6):679-80. Epub 2014/05/24. doi: 10.1007/s00247-014-2971-7. PubMed PMID: 24854937.
8. Bélard S, Tamarozzi F, Bustinduy AL, Wallrauch C, Grobusch MP, Kuhn W, et al. Point-of-Care Ultrasound Assessment of Tropical Infectious Diseases--A Review of Applications and Perspectives. Am J Trop Med Hyg. 2016;94(1):8-21. Epub 2015/09/28. doi: 10.4269/ajtmh.15-0421. PubMed PMID: 26416111.
9. Bélard S, Heller T, Orie V, Heuvelings CC, Bateman L, Workman L, et al. Sonographic Findings of Abdominal Tuberculosis in Children With Pulmonary Tuberculosis. Pediatr Infect Dis J. 2017;36(12):1224-6. Epub 2017/03/24. doi: 10.1097/inf.0000000000001590. PubMed PMID: 28333710; PubMed Central PMCID: PMCPMC5610051.
10. Belard S, Heuvelings C, Heller T, Andronikou S, Grobusch M, Zar H. Point-of-care Ultrasound for Pulmonary and Extrapulmonary Tuberculosis in Children. The Pediatric Infectious Disease Journal. 2019;38:e110. doi: 10.1097/INF.0000000000002243.
11. Ben Saad-Baouab S, Hantous S, Daghfous H, Ben Miled K, Tritar F. Contribution of imaging in the management of resistant tuberculosis. Tunis Med. 2019;97(3):445-54. Epub 2019/11/16. PubMed PMID: 31729719.
12. Bhalla AS, Goyal A, Guleria R, Gupta AK. Chest tuberculosis: Radiological review and imaging recommendations. Indian J Radiol Imaging. 2015;25(3):213-25. doi: 10.4103/0971-3026.161431. PubMed PMID: 26288514.
13. Brindle HE, Allain TJ, Kampondeni S, Kayange N, Faragher B, Bates I, et al. Utilization of ultrasound in medical inpatients in Malawi. Transactions of The Royal Society of Tropical Medicine and Hygiene. 2013;107(7):405-10. doi: 10.1093/trstmh/trt034.
14. Brunetti E, Heller T, Richter J, Kaminstein D, Youkee D, Giordani MT, et al. Application of Ultrasonography in the Diagnosis of Infectious Diseases in Resource-Limited Settings. Curr Infect Dis Rep. 2016;18(2):6. Epub 2016/01/20. doi: 10.1007/s11908-015-0512-7. PubMed PMID: 26781324.
15. Buonsenso D, Pezza L, Valentini P. Utility of Point-of-care Ultrasound in Children With Pulmonary Tuberculosis. Pediatr Infect Dis J. 2018;37(11):e280-e1. Epub 2018/10/12. doi: 10.1097/inf.0000000000002086. PubMed PMID: 30308605.
16. Buonsenso D, Curatola A, Valentini P, Scialanga B, Toma P, Musolino AM. Chest ultrasound findings in children with confirmed pulmonary tuberculosis in low tuberculosis incidence country. Pediatr Pulmonol. 2019;54(9):1348-50. Epub 2019/05/21. doi: 10.1002/ppul.24362. PubMed PMID: 31106507.

17. Burleson SL, Pigott DC, Gullett JP, Greene C, Gibson CB, Irvine S, et al. Point-of-care ultrasound in resource-limited settings: the PURLS fellowship. The Ultrasound Journal. 2020;12(1):14. doi: 10.1186/s13089-020-00159-6.
18. Canan T, Hoffman RM, Schooley A, Boas Z, Schwab K, Kahn D, et al. M#1045: Training Course in Focused Assessment with Sonography for HIV/TB in HIV Prevalent Medical Centers in Malawi. J Glob Radiol. 2018;4(1). Epub 2018/01/01. doi: 10.7191/jgr.2018.1045. PubMed PMID: 31633008; PubMed Central PMCID: PMCPMC6800233.
19. Chesov DB, V. Imaging for diagnosis and management. ERS Monograph. 2018;9781849841009:116-36.
20. Di Gennaro F, Pisani L, Veronese N, Pizzol D, Lippolis V, Saracino A, et al. Potential Diagnostic Properties of Chest Ultrasound in Thoracic Tuberculosis-A Systematic Review. Int J Environ Res Public Health. 2018;15(10). Epub 2018/10/17. doi: 10.3390/ijerph15102235. PubMed PMID: 30322009; PubMed Central PMCID: PMCPMC6210728.
21. dos Reis LVT, Faria LF, Corrêa TJ, Fernandes GDS, Corrêa RDS, Thiago Thomaz M, et al. Correlation Between Pulmonary Ultrasonography and Tuberculous Pleural Effusion. B66 WHAT'S NEW IN VASCULAR AND PLEURAL DISEASE. American Thoracic Society International Conference Abstracts: American Thoracic Society; 2018. p. A3956-A.
22. Elmahalawy II, Doha NM, Ebeid OM, Abdel-Hady MA, Saied O. Role of thoracic ultrasound in diagnosis of pulmonary and pleural diseases in critically ill patients. Egyptian Journal of Chest Diseases and Tuberculosis. 2017;66(2):261-6. doi: https://doi.org/10.1016/j.ejcdt.2016.10.005.
23. Elnaem WH, Tammam HM, Zidan MA, Mahmoud MI. The relative efficacy of chest ultrasonography in comparison to other diagnostic modalities in the evaluation of dyspneic patient. Egyptian Journal of Chest Diseases and Tuberculosis. 2017;66(1):165-8. doi: https://doi.org/10.1016/j.ejcdt.2016.12.005.
24. Fentress M, Heyne TF, Barron KR, Jayasekera N. Point-of-Care Ultrasound in Resource-Limited Settings: Common Applications. South Med J. 2018;111(7):424-33. Epub 2018/07/07. doi: 10.14423/smj.0000000000000827. PubMed PMID: 29978229.
25. Gati S, Chetty R, Wilson D, Achkar JM. Utilization and Clinical Value of Diagnostic Modalities for Tuberculosis in a High HIV Prevalence Setting. Am J Trop Med Hyg. 2018;99(2):317-22. Epub 2018/06/07. doi: 10.4269/ajtmh.17-0965. PubMed PMID: 29893198.
26. Gavrila IL, Badea RI, Jude C, Socaciu MA, Comsa M, Badea AF. Ultrasound as the first imaging method in severe lung disease. Considerations about a case of pulmonary tuberculosis and review of the literature. Med Ultrason. 2020;22(1):102-4. Epub 2020/02/26. doi: 10.11152/mu-1890. PubMed PMID: 32096796.
27. Giordani MT, Heller T. Role of ultrasound in the diagnosis of tuberculosis. European Journal of Internal Medicine. 2019;66:27-8. doi: https://doi.org/10.1016/j.ejim.2019.07.002.
28. Heller T, Wallrauch C, Lessells RJ, Goblirsch S, Brunetti E. Short course for focused assessment with sonography for human immunodeficiency virus/tuberculosis: preliminary results in a rural setting in South Africa with high prevalence of human immunodeficiency virus and tuberculosis. Am J Trop Med Hyg. 2010;82(3):512-5. Epub 2010/03/09. doi: 10.4269/ajtmh.2010.09-0561. PubMed PMID: 20207884; PubMed Central PMCID: PMCPMC2829920.
29. Heller T, Wallrauch C, Brunetti E, Giordani MT. Changes of FASH ultrasound findings in TB-HIV patients during anti-tuberculosis treatment. Int J Tuberc Lung Dis. 2014;18(7):837-9. Epub 2014/06/07. doi: 10.5588/ijtld.13.0029. PubMed PMID: 24902561.
30. Heller T, Mtemang'ombe EA, Huson MA, Heuvelings CC, Bélard S, Janssen S, et al. Ultrasound for patients in a high HIV/tuberculosis prevalence setting: a needs assessment and review of focused applications for Sub-Saharan Africa. Int J Infect Dis. 2017;56:229-36. Epub 2016/11/12. doi: 10.1016/j.ijid.2016.11.001. PubMed PMID: 27836795.
31. Henriquez-Camacho C, Garcia-Casasola G, Guillén-Astete C, Losa J. Ultrasound for the diagnosis of infectious diseases: Approach to the patient at point of care and at secondary level. J Infect. 2015;71(1):1-8. Epub 2015/03/24. doi: 10.1016/j.jinf.2015.03.003. PubMed PMID: 25797569.

32. Heuvelings CC, Bélard S, Janssen S, Wallrauch C, Grobusch MP, Brunetti E, et al. Chest ultrasonography in patients with HIV: a case series and review of the literature. Infection. 2016;44(1):1-10. Epub 2015/05/14. doi: 10.1007/s15010-015-0780-z. PubMed PMID: 25972115.
33. Heuvelings CB, S.; Andronikou, S.; Grobusch, M.; Zar, H. Mediastinal ultrasound versus chest X-ray for the detection of lymphadenopathy in children with suspected pulmonary tuberculosis. Pediatric radiology Conference: 60th annual meeting of the society for pediatric radiology, SPR 2017. 2017;47(1):1-296. doi: 10.1007/s00247-017-3809-x.
34. Heuvelings CCB, S.; Andronikou, S.; Jamieson-Luff, N.; Grobusch, M. P.; Zar, H. J. Point-of-care chest ultrasound in South African children with suspected pulmonary tuberculosis. Tropical Medicine & International Health. 2017;22:42-.
35. Heuvelings CCB, S.; Andronikou, S.; Moodley, H.; Jamieson-Luff, N.; Grobusch, M. P.; Zar, H. J. Chest Ultrasound versus X-ray for Pulmonary Tuberculosis in South African Children. American Journal of Tropical Medicine and Hygiene. 2017;97(5):244-.
36. Heuvelings CC, Bélard S, Andronikou S, Lederman H, Moodley H, Grobusch MP, et al. Chest ultrasound compared to chest X-ray for pediatric pulmonary tuberculosis. Pediatric pulmonology. 2019;54(12):1914-20. Epub 2019/09/01. doi: 10.1002/ppul.24500. PubMed PMID: 31475477.
37. Hunter L, Bélard S, Janssen S, van Hoving DJ, Heller T. Miliary tuberculosis: sonographic pattern in chest ultrasound. Infection. 2016;44(2):243-6. Epub 2015/12/15. doi: 10.1007/s15010-015-0865-8. PubMed PMID: 26661658.
38. Kahn D, Pool K-L, Phiri L, Chibwana F, Schwab K, Longwe L, et al. Diagnostic Utility and Impact on Clinical Decision Making of Focused Assessment With Sonography for HIV-Associated Tuberculosis in Malawi: A Prospective Cohort Study. Glob Health Sci Pract. 2020;8(1):28-37. doi: 10.9745/GHSP-D-19-00251. PubMed PMID: 32041772.
39. Kaminstein D, Kuhn WT, Huang D, Burleson SL. Perspectives on Point-of-Care Ultrasound Use in Pediatric Tropical Infectious Disease. Clinical Pediatric Emergency Medicine. 2019;20(2):128-40. doi: https://doi.org/10.1016/j.cpem.2019.06.003.
40. Mirijello A, De Cosmo S, Sperandeo M. Lung ultrasonography in pulmonary tuberculosis: Integrating chest radiology? Eur J Intern Med. 2019;69:e17-e8. Epub 2019/08/04. doi: 10.1016/j.ejim.2019.07.023. PubMed PMID: 31375254.
41. Montuori M, Cogliati C. Lung ultrasonography in pulmonary tuberculosis: Integrating chest radiology? Authors' reply. European Journal of Internal Medicine. 2019;69:e19-e20. doi: https://doi.org/10.1016/j.ejim.2019.08.021.
42. Moseme T, Andronikou S. Through the eye of the suprasternal notch: point-of-care sonography for tuberculous mediastinal lymphadenopathy in children. Pediatr Radiol. 2014;44(6):681-4. Epub 2014/05/24. doi: 10.1007/s00247-014-2890-7. PubMed PMID: 24854938.
43. Ndege R, Weisser M, Elzi L, Diggelmann F, Bani F, Gingo W, et al. Sonography to Rule Out Tuberculosis in Sub-Saharan Africa: A Prospective Observational Study. Open Forum Infectious Diseases. 2019;6(4). doi: 10.1093/ofid/ofz154.
44. Ndege R, Ngome O, Bani F, Temba Y, Wilson H, Vanobberghen F, et al. Ultrasound in managing extrapulmonary tuberculosis: a randomized controlled two-center study. BMC Infect Dis. 2020;20(1):349. Epub 2020/05/18. doi: 10.1186/s12879-020-05073-9. PubMed PMID: 32414338; PubMed Central PMCID: PMCPMC7226714.
45. Pomykala K, Desai I, Jardon M, Naik P, Pool K-L. Imaging of Tuberculosis in Resource-Limited Settings. Current Radiology Reports. 2019;7(8):23. doi: 10.1007/s40134-019-0335-7.
46. Pool KL, Heuvelings CC, Bélard S, Grobusch MP, Zar HJ, Bulas D, et al. Technical aspects of mediastinal ultrasound for pediatric pulmonary tuberculosis. Pediatr Radiol. 2017;47(13):1839-48. Epub 2017/08/31. doi: 10.1007/s00247-017-3954-2. PubMed PMID: 28852808.
47. van Hoving DJ, Lamprecht HH, Stander M, Vallabh K, Fredericks D, Louw P, et al. Adequacy of the emergency point-of-care ultrasound core curriculum for the local burden of disease in South Africa. Emerg Med J. 2013;30(4):312-5. Epub 2012/05/18. doi: 10.1136/emermed-2012-201358. PubMed PMID: 22593261.

48. Van Hoving DJ, Kenge AP, Maartens G, Meintjes G. Point-of-Care Ultrasound Predictors for the Diagnosis of Tuberculosis in HIV-Positive Patients Presenting to an Emergency Center. J Acquir Immune Defic Syndr. 2020;83(4):415-23. Epub 2020/01/07. doi: 10.1097/qai.0000000000002279. PubMed PMID: 31904699.
